# An optimization method for implantation parameters of individualized TKA tibial prosthesis based on finite element analysis and orthogonal experimental design

**DOI:** 10.1186/s12891-020-3189-5

**Published:** 2020-03-12

**Authors:** Yuefu Dong, Zhen Zhang, Wanpeng Dong, Guanghong Hu, Bing Wang, Zhifang Mou

**Affiliations:** 1 Department of Orthopedics, The Affiliated Lianyungang Hospital of Xuzhou Medical University/the First People’s Hospital of Lianyungang, Lianyungang, China; 2 School of Materials Engineering, Shanghai University of Engineering Science, Shanghai, China; 3 Institute of Plasticity Forming Technology & Equipment, Shanghai Jiao Tong University, Shanghai, China; 4 Department of Critical Care Medicine, The Affiliated Lianyungang Hospital of Xuzhou Medical University/the First People’s Hospital of Lianyungang, No.8 Lingzhou East Road, Haizhou District, Lianyungang, 222000 China

**Keywords:** Total knee arthroplasty, Prosthesis, Implantation parameters, Optimization method, Finite element analysis, Orthogonal experimental design

## Abstract

**Background:**

Individualized and accurate implantation of a tibial prosthesis during total knee arthroplasty (TKA) can assist in uniformly distributing the load and reducing the polyethylene wear to obtain a long-term prosthetic survival rate, but individualized and accurate implantation of a tibial prosthesis during TKA remains challenging. The purpose of this study was to optimize and individualize the positioning parameters of a tibial prosthesis to improve its accurate implantation using a new method of finite element analysis in combination with orthogonal experimental design.

**Methods:**

Ten finite element models of TKA knee joint were developed to optimize the implantation parameters (varus angle, posterior slope angle, and external rotation angle) of tibial prosthesis to reduce the peak value of the contact pressure on the polyethylene liner according to the method of finite element analysis in combination with orthogonal experimental design. The influence of implantation parameters on the peak value of the contact pressure on the polyethylene liner was evaluated based on a range analysis in orthogonal experimental design.

**Results:**

The optimal implantation parameters for tibial prosthesis included 0° varus, 1° posterior slope, and 4° external rotation. Under these conditions, the peak value of the contact pressure on the polyethylene liner remained the smallest (16.37 MPa). Among the three parameters that affect the peak value of the contact pressure, the varus angle had the greatest effect (range = 6.70), followed by the posterior slope angle (range = 2.36), and the external rotation angle (range = 2.15).

**Conclusions:**

The optimization method based on finite element analysis and orthogonal experimental design can guide the accurate implantation of the tibial prosthesis, reducing the peak value of the contact pressure on the polyethylene liner. This method provides new insights into the TKA preoperative plan and biomechanical decision-making for accurately implanting TKA prosthesis.

## Background

Total knee arthroplasty (TKA) is one of the main procedures to treat end-stage knee diseases, and can effectively relieve pain and improve function as well as the quality of life of the patients. The 10-year survival rate for individual prostheses is higher than 95% [1, 2]. Individualized and accurate bone cuts and implantation of prosthetic components are considered as basic technical requirements of TKA, with the aim of restoring the neutral mechanical alignment of lower extremity, promoting uniform distribution of load in the knee joint, reducing wear of the polyethylene liner, and prolonging the survival of the TKA prosthesis [3, 4]. Implant malalignment causes nonuniform load distribution and stress concentration, leading to the wear of the polyethylene liner, instability of the joint, and osteolysis, and finally requiring revision surgery [5]. Accurate implantation of tibial prosthesis can effectively neutralize the mechanical alignment of the lower extremity. Owing to the relatively high frequency of tibial osteolysis, the uncertainty of tibial rotation references, and the significant influence of tibial prosthesis alignment on the load distribution in the knee joint, orthopedic surgeons have to pay special attention to the accuracy of tibial prosthesis implantation during TKA [4].

Currently, there are many preoperative evaluation methods for guiding the implantation of the tibial prosthesis, which assists in restoring the neutral mechanical alignment of the lower extremity. Clinically, implantation parameters such as tibial resection angle (varus/valgus angle) in the coronal plane, posterior slope angle in the sagittal plane, and external rotation angle in the axial plane are determined through imaging data of the lower extremity and help in guiding the intraoperative implantation of tibial prosthesis during TKA [6]. The application of computer-navigated TKA and patient-specific instrumentation improves the accuracy of implantation of TKA prosthesis and achieves an ideal position of the postoperative lower extremity mechanical axis [7, 8]. Although these preoperative plans based on imaging data can help restore the neutral mechanical alignment of the lower extremity, they fail to visually display the load and stress distribution of TKA knee and/or reliably predict the survival of TKA prostheses.

Therefore, how to determine the optimal implantation parameters before undergoing TKA based on the intra-articular load distribution in an individual patient and uniform distribution of the load are difficult to achieve by conventional preoperative planning solely using imaging data of the lower extremity. Finite element analysis is regarded as an effective method to solve this issue by optimizing the implantation parameters of the tibial prosthesis [4]. Stan et al. analyzed the influence of different varus angles of tibial prosthesis on the contact pressure on the polyethylene liner by the finite element method. The results revealed that the larger the tibial prosthesis varus angle was, the greater the peak value of the contact pressure on the polyethylene liner was, and the more likely wear of the polyethylene liners would occur [9]. Osano et al. found that internal or external rotation of tibial prosthesis caused change in the peak value of the contact pressure on the polyethylene liner, and the greater the internal rotation angle of the prosthesis was, the greater was the change in the peak value of the contact pressure on the polyethylene liner [10]. Shen et al. analyzed the effect of the posterior slope angle of tibial prosthesis on the peak value of contact stress on the polyethylene liner. The results showed that the posterior slope of 3° or 6° of tibial prosthesis had a relatively small peak value of the contact stress [11]. The above studies analyzed the effect of only a single tibial prosthesis implantation parameter on the knee contact mechanics, which demonstrated a certain clinical significance for tibial prosthesis implantation. Nevertheless, it cannot be used as a detailed preoperative plan to guide the individualized and accurate implantation of the tibial prosthesis. There are no studies so far that reported the use of finite element analysis as a preoperative plan to guide individualized tibial prosthesis implantation. The possible reason for this is that several TKA finite element models are required, limiting its clinical application widely. Orthogonal experimental design can effectively reduce the number of TKA finite element models, improve the efficiency of calculation and analysis, and shorten the preoperative planning time. Currently, there are no studies that report the application of orthogonal experimental design in TKA finite element analysis as preoperative planning.

Therefore, this study aimed to conduct the simulated implantation of TKA prostheses and finite element analysis based on a previously constructed knee joint model, and optimize the individualized tibial prosthesis implantation parameters to reduce the peak value of the contact pressure on the upper surface of the polyethylene liner and its wear, and extend the life of the prosthesis by combining with orthogonal experimental design. Meanwhile, the degree of influence of different implantation parameters on the peak value of the contact pressure on polyethylene liner was determined to guide in effectively implanting the tibial prosthesis. The optimization method for TKA implantation parameters based on finite element analysis and orthogonal experimental design was established in this study to explore TKA preoperative planning, which should promote the individualized and accurate implantation of TKA prosthesis.

## Methods

### Finite element model of TKA knee joint

#### Construction of the three-dimensional (3D) model of TKA prosthesis

**Fig. 1:**
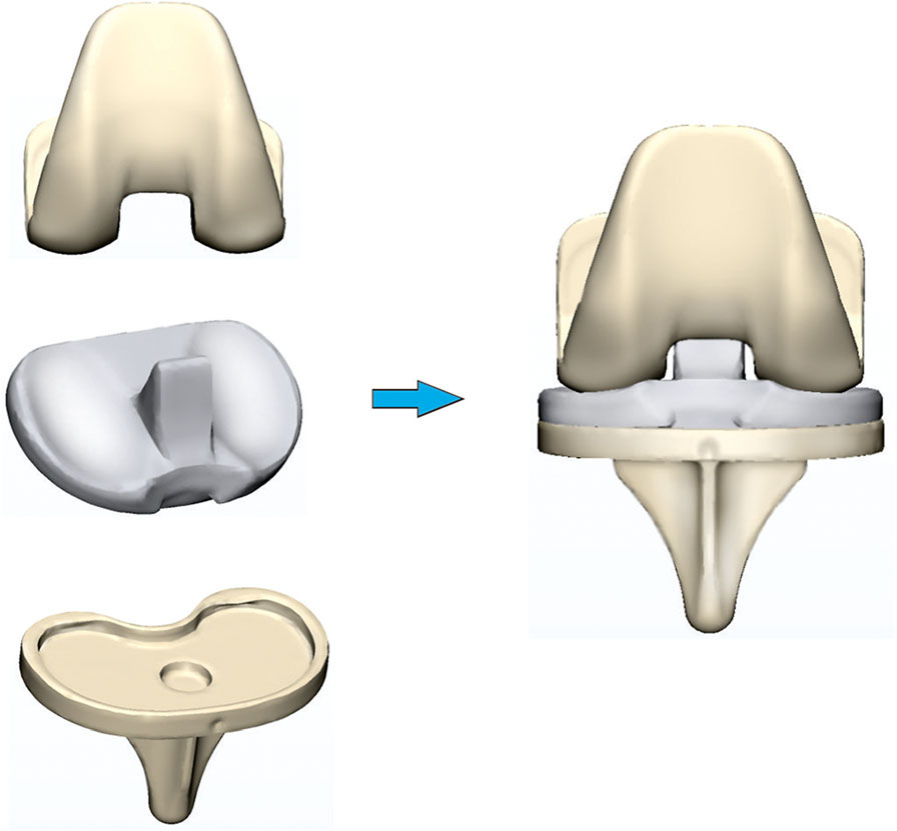
3D model of TKA prosthesis

This study was approved by the ethics committee of our hospital (No. 2016008), and informed consent was signed by the volunteer and his family members. Measurements of the distal femur and proximal tibia were obtained based on knee image data, including the anterior and posterior mediolateral length of the distal femur, the medial and lateral anteroposterior diameter of the distal femur, the mediolateral width of the proximal tibial, and the medial and lateral anteroposterior length of the medial and lateral tibial plateau. Appropriate sizes (size 5 femoral component, size 4 tibial tray, and 9-mm polyethylene liner) of a cemented, posterior-stabilized knee prosthesis system (Smith and Nephew, Memphis, TN, USA) were selected based on the aforementioned measurements and scanned by a laser scanner (Renishaw, London, UK) to obtain the .stl files of the TKA prosthesis. The .stl files were then imported into the Mimics 19.0 (Materialise, Leuven, Belgium) software to construct a 3D model of the TKA prosthesis (Fig. 1).

#### Simulated implantation of the TKA prosthesis

**Fig. 2:**
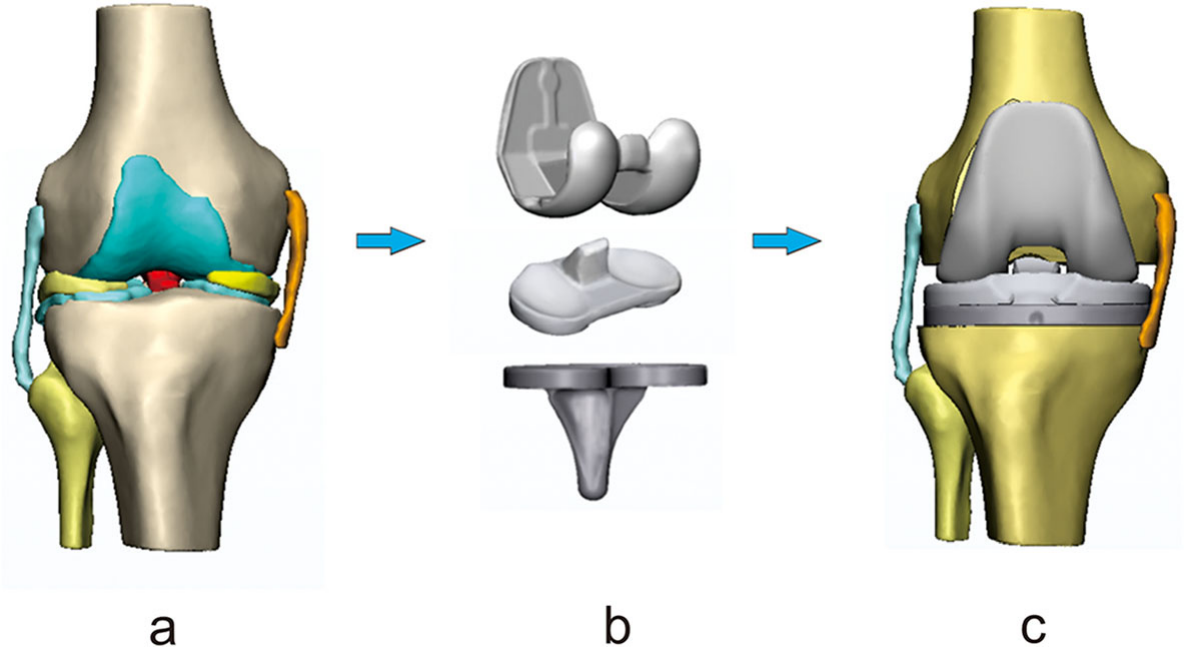
Simulated implantation of TKA prosthesis. **a** 3D model of the knee joint; **b** 3D model of TKA prosthesis; **c** Overall 3D model of the TKA knee joint

The simulated implantation of the TKA prosthesis was performed based on a previously constructed and verified 3D knee joint model (Fig. 2a) [12, 13]. The full-length weight-bearing anteroposterior radiograph of the leg of a 24-year-old male volunteer (body mass index of 22.9 kg/m^2^, hip-knee-ankle angle of 180°, mechanical lateral distal femoral angle of 88°, and medial proximal tibial angle of 87°) was obtained to determine the implantation parameters of tibial prosthesis according to the operational principles of the primary TKA, i.e., 0° varus (i.e., the proximal tibial cut was perpendicular to the tibial mechanical axis in the coronal plane), 1° posterior slope (i.e., the angle between the proximal tibial cut surface and the anatomical axis of tibia in the sagittal plane), and 3° external rotation (i.e., the posterior margin line of the tibial tray was rotated 3° externally with respect to the posterior margin line of the proximal tibial cut in the axial plane) were taken as the tibial prosthesis implantation parameters. The constructed TKA knee joint model was used as the basic model. A simulated osteotomy on the tibia and femur was performed using the Mimics software. According to the measurements, a size 5 femoral and a size 4 tibial component, 9-mm polyethylene liner, and 1-mm resection plate were used to perform a simulated osteotomy and prosthetic implantation. A 1-mm space was maintained between the prosthesis and the bone cuts in order to implant a 1-mm-thick bone cement layer. The tibial prosthesis was implanted on the proximal tibia, and the femoral prosthesis was accurately matched with the tibial prosthesis and implanted in the distal end of the femur. The polyethylene liner was implanted to match with the tibial prosthesis to obtain an overall 3D model of the TKA knee joint (Fig. 2c).

#### Construction of the finite element model of the TKA knee joint

**Fig. 3:**
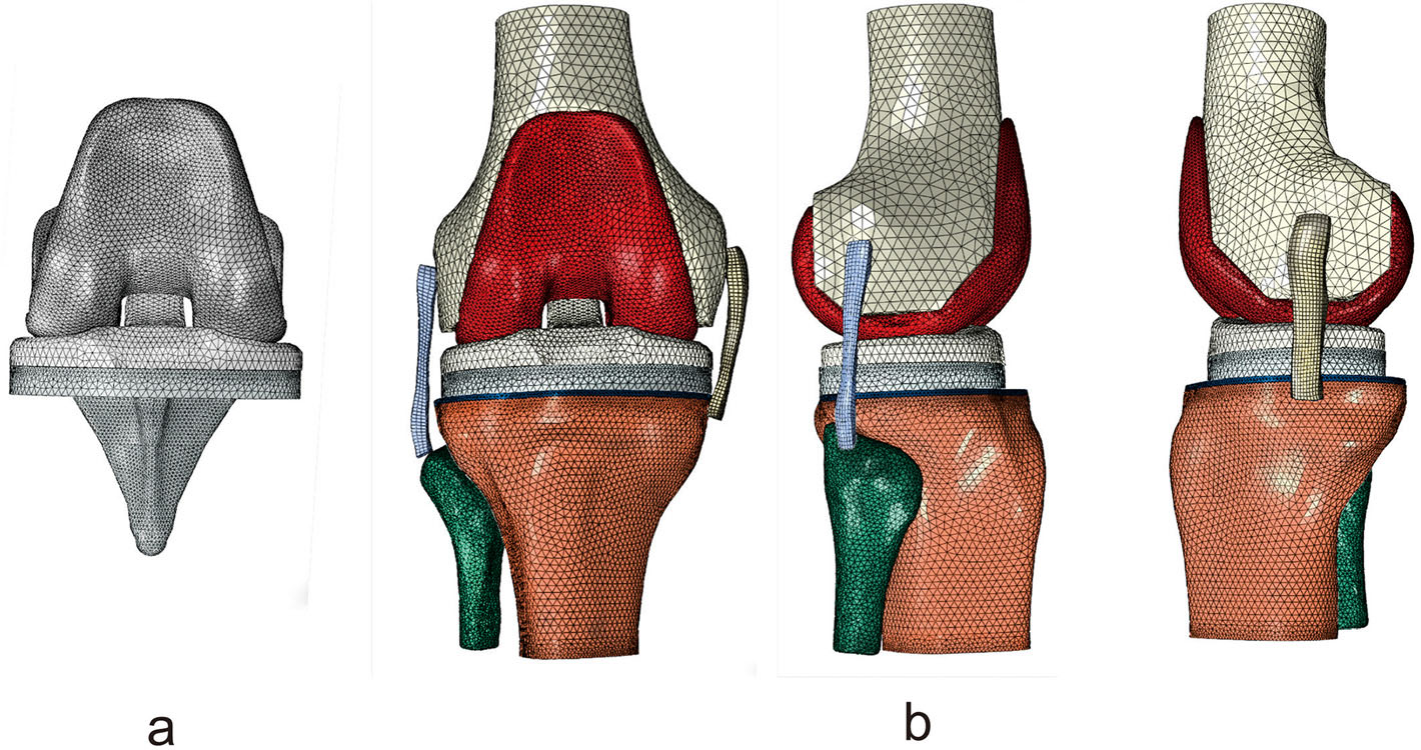
3D finite element model of the TKA knee joint. **a** 3D finite element model of TKA prosthesis; **b** Overall 3D finite element model of the TKA knee joint

The anatomical structures of the knee joint and TKA prosthetic components were imported into the Hypermesh 14.0 software (Altair, Clifton Park, NY, USA) in the form of a .stl file. Bone cement with a thickness of 1 mm was generated between the bone cuts and the prosthesis, and the mesh was generated to construct a 3D finite element model of TKA knee joint that consists of the femur, tibia, fibula, medial, and lateral collateral ligaments, tibial prosthesis, femoral prosthesis, polyethylene liner, and bone cement layers (Fig. 3b).

#### Material properties, boundary conditions, and loading

The finite element analysis of the TKA knee was carried out using Abaqus 6.14 (Simulia, Providence, RI, USA). The medial and lateral collateral ligaments of the knee joint were defined as an isotropic hyperelastic material based on an incompressible Neo-Hookean behavior containing the energy density function equation:

1 $$ \uppsi ={\mathrm{C}}_1\left(\overset{\sim }{{\mathrm{I}}_1}-3\right) $$

where C_1_ is the initial shear modulus, and $\overset{\sim }{{\mathrm{I}}_1}$ is the first modified invariant for the right Cauchy-Green strain tensor [14]. The C_1_ of the lateral and medial collateral ligaments were defined as 6.06 and 6.43, respectively, and the number of elements and nodes were (982, 1660) and (1342, 2122), respectively [14]. The bone structures and the TKA prosthetic components were defined as isotropic linear elastic materials (Table 1).

**Table 1:** TKA knee material properties, number of elements and nodes

	Elastic modulus (MPa)	Poisson’s ratio	Number of elements	Number of nodes
Cortical bone	16,600	0.3	23,231	7976
Cancellous bone	2400	0.3	54,532	14,892
Femoral prosthesis	210,000	0.3	88,966	21,267
Tibial prosthesis	117,000	0.3	39,785	9496
Polyethylene liner	685	0.4	29,731	7856
Bone cement layer	3000	0.3	31,754	10,243

**Fig. 4:**
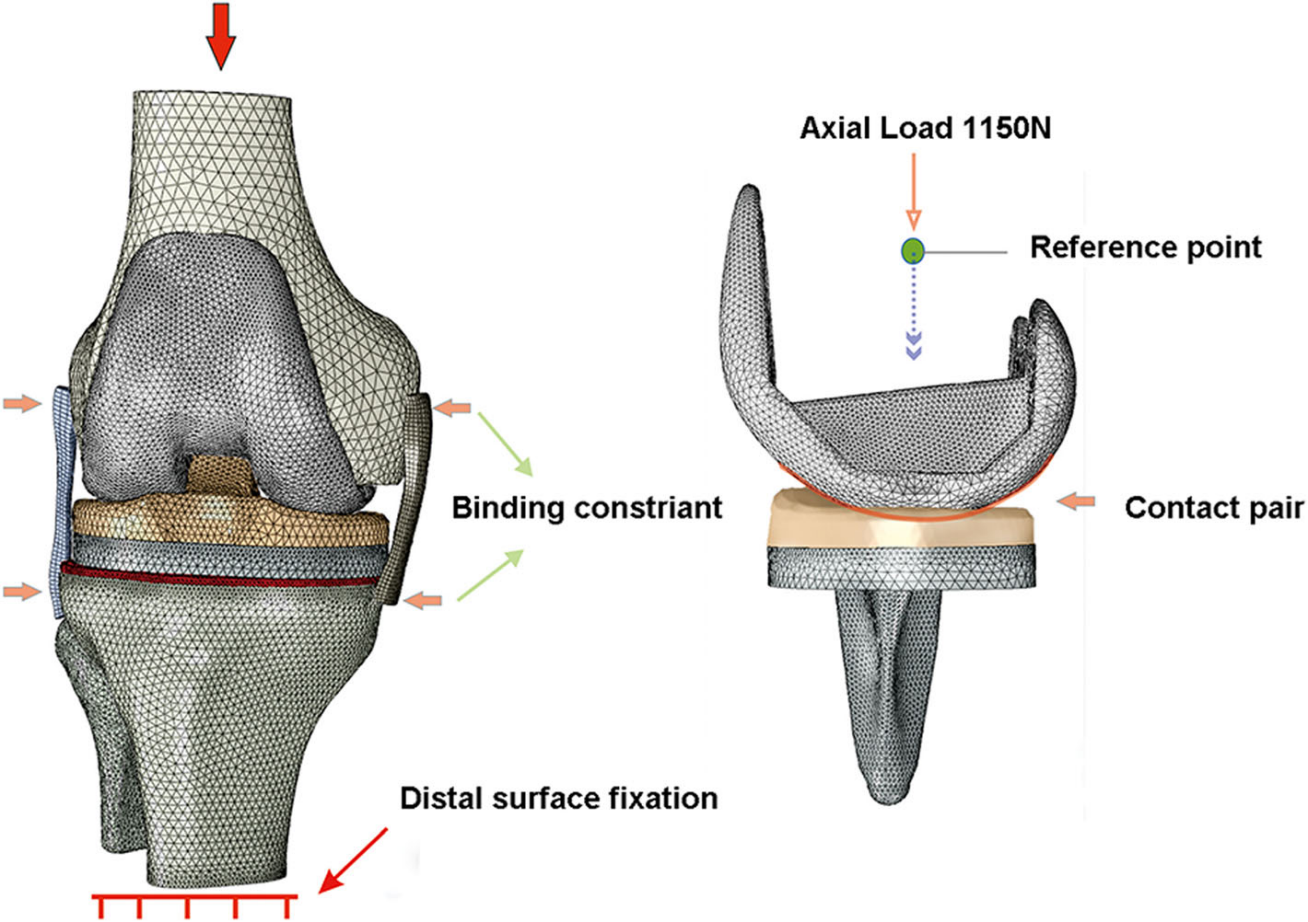
Boundary conditions and loading of the finite element analysis

The boundary conditions of finite element analysis were as follows (Fig. 4):

1. At 0° flexion position, the flexion and extension of the femur were limited, and the remaining two rotations and three translations were unconstrained, and the distal tibia and fibula were limited in all their rotations and translations.
2. Binding constraints were applied between the cortical bone and the cancellous bone, the prosthesis and the bone cement layer, the bone cement layer and the bone cutting surfaces, and the polyethylene liner and the tibial prosthesis.
3. Binding constraints were applied in the connection between the medial and lateral collateral ligaments and the bones.
4. Nonlinear contact was selected, and the friction coefficient was set as 0.04 [15]. The contact pair included the femoral prosthesis and the polyethylene liner. Small sliding and finite sliding were set as the contact conditions.

A vertical compressive load of 1150 N (along the Z-axis, about twice the body weight; Fig. 4) was applied onto the midpoint of the transepicondylar axis of the femur to simulate the load of the gait cycle at 0° flexion position [12, 13]. The variable of the peak value of the contact pressure on the upper surface of the polyethylene liner was observed.

### Orthogonal experimental design and optimization analysis

Three factors were selected as optimization parameters of the tibial prosthesis implantation. Three implantation parameters, A (varus angle, varus for positive, and valgus for negative), B (posterior slope angle), and C (external rotation angle) were taken as orthogonal experimental factors. All three levels were selected for each factor (Table 2), and an orthogonal table L_9_ (3^4^) was selected. The basic model was re-adjusted by using the Hypermesh software to create different finite element models of the TKA knee joint by meeting the requirements of factor combinations in the orthogonal experimental design.

**Table 2:** Orthogonal experimental design

Level	Experimental factor		
	Varus angle A (°)	Posterior slope angle B (°)	External rotation angle C (°)
1	0	1	3
2	3	2	4
3	−3	3	5

According to the orthogonal experimental design, nine groups of experimental level combinations (the combinations of implantation parameters) were determined, and nine TKA finite element models were established. For performing finite element analysis, similar material properties, boundary conditions, and loads were set for each model. Through orthogonal experimental optimization analysis, the order of influence of the tibial implantation parameters on the peak value of the contact pressure on the upper surface of the polyethylene liner was determined, and an optimal combination of TKA implantation parameters with minimum peak value was then obtained.

### Verification of the optimized implantation parameters derived from the orthogonal experimental design

Based on the implantation parameters of the tibial prosthesis as optimized by the orthogonal experimental analysis, a finite element model of TKA knee was re-constructed for carrying out finite element analysis. Besides, distribution of the peak contact pressure on the polyethylene liner was analyzed and compared with other models to verify whether the orthogonal experimental optimization analysis could reduce the peak value of contact pressure and reduce the wear of the polyethylene liner.

## Results

### Distribution of the contact pressure on the polyethylene liner

**Fig. 5:**
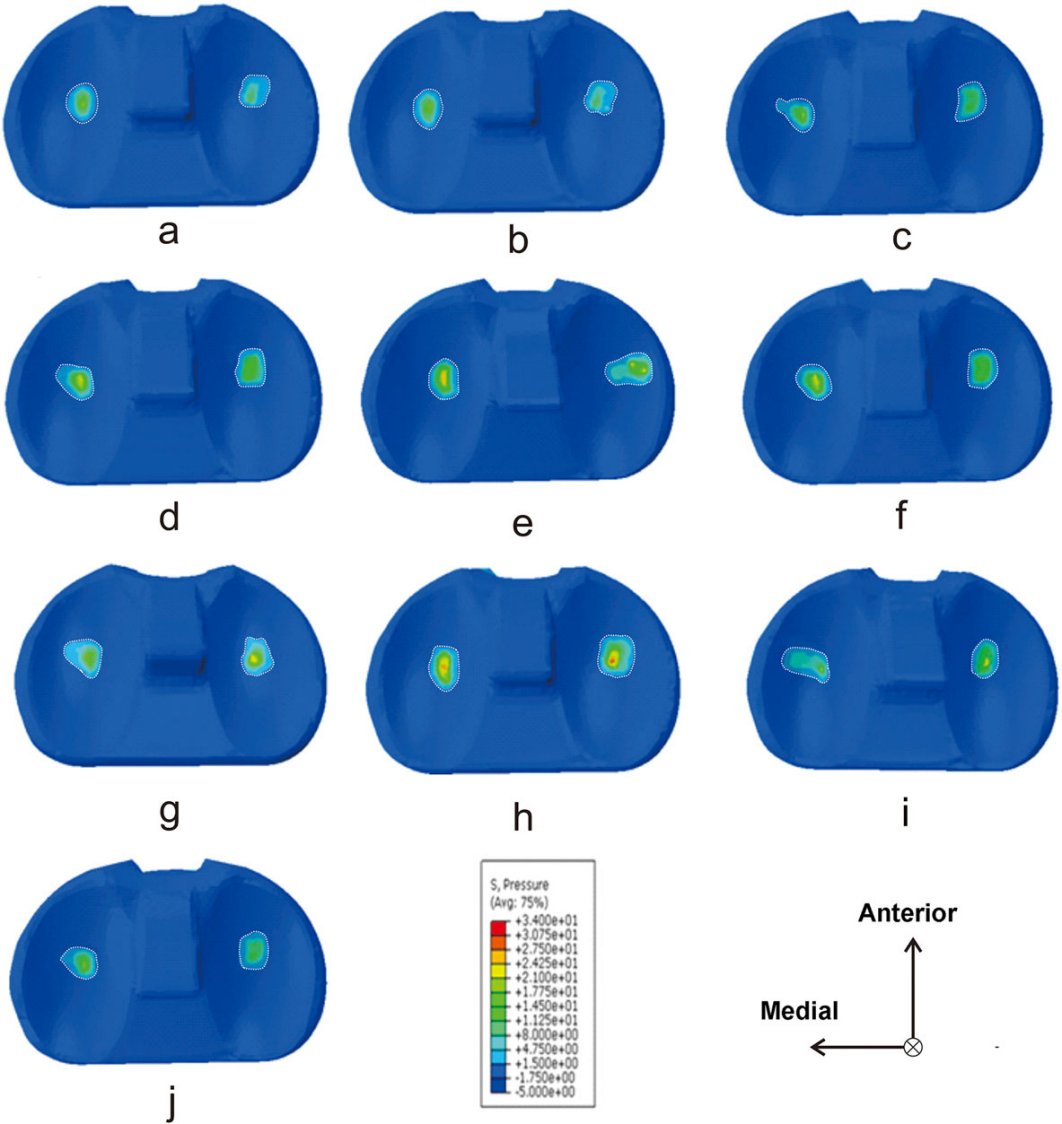
Distribution of the contact pressure on the polyethylene liner. **a** Model of the A_1_B_1_C_1_ group; **b** Model of the A_1_B_2_C_2_ group; **c** Model of the A_1_B_3_C_3_ group; **d** Model of the A_2_B_1_C_2_ group; **e** Model of the A_2_B_2_C_3_ group; **f** Model of the A_2_B_3_C_1_ group; **g** Model of the A_3_B_1_C_3_ group; **h** Model of the A_3_B_2_C_1_ group; **i** Model of the A_3_B_3_C_2_ group; and **j** Model of the A_1_B_1_C_2_ group (optimal group)

Through finite element analysis, the peak value of the contact pressure on the upper surface of the polyethylene liner of each model is shown in Table 3. Among the nine models, the highest peak value of the contact pressure was observed to be 29.73 MPa (group A_3_B_2_C_1_, Fig. 5h), which was located in the lateral compartment. The lowest value was shown as 17.10 MPa (group A_1_B_2_C_2_, Fig. 5b), which was located in the medial compartment. The maximal difference in the peak value of the contact pressure between the lateral and medial compartments was 6.52 MPa (group A_3_B_3_C_2_, Fig. 5i). According to these results, minor variations in implantation parameters of the tibial prosthesis could lead to a relatively large change in contact pressure (the maximum change value was 12.63 MPa), indicating the importance of individualized and accurate implantation of the tibial prosthesis.

**Table 3:** Experimental level combinations of orthogonal experimental design

Model	Experimental level combination	Varus angle (°)	Posterior slope angle (°)	External rotation angle (°)	Peak value of the contact pressure (MPa)
1	A_1_B_1_C_1_	0	1	3	18.99
2	A_1_B_2_C_2_	0	2	4	17.10
3	A_1_B_3_C_3_	0	3	5	20.46
4	A_2_B_1_C_2_	3	1	4	22.18
5	A_2_B_2_C_3_	3	2	5	24.10
6	A_2_B_3_C_1_	3	3	3	21.26
7	A_3_B_1_C_3_	−3	1	5	22.68
8	A_3_B_2_C_1_	−3	2	3	29.73
9	A_3_B_3_C_2_	−3	3	4	24.25

### Optimization results of the orthogonal experimental design

Range analysis of the peak value of the contact pressure on the polyethylene liners of the nine knee models was performed. Table 4 presented the results of the orthogonal experiment, in which K_ji_ represents the mean of the peak value of the contact pressure for the experimental factor *j* at level *i*.

**Table 4:** Optimization of the results of the orthogonal experimental design

Model	Experimental factor *j* (*j* = 1,2,3,4)				Peak value of the contact pressure (MPa)
	A	B	C	D	
1	1	1	1	1	18.99
2	1	2	2	2	17.10
3	1	3	3	3	20.46
4	2	1	2	3	22.18
5	2	2	3	1	24.10
6	2	3	1	2	21.26
7	3	1	3	2	22.68
8	3	2	1	3	29.73
9	3	3	2	1	24.25
K_j1_	18.85	21.28	23.33		
K_j2_	22.51	23.64	21.18		
K_j3_	25.55	21.99	22.41		
Range R_j_	6.70	2.36	2.15		
Ranking	A > B > C				
Optimal level	A_1_	B_1_	C_2_		

The range was calculated by using the formula: ${\mathrm{R}}_{\mathrm{j}}=\underset{1\le \mathrm{i}\le \mathrm{m}}{\max }{\mathrm{k}}_{\mathrm{j}\mathrm{i}}-\underset{1\le \mathrm{i}\le \mathrm{m}}{\min }{\mathrm{k}}_{\mathrm{j}\mathrm{i}},$ where *Rj* represents the range of experimental factor *j*, and *m* is the number of levels selected by a factor. The greater the range of factors, the more significant is their influence on the results. According to the results of the range analysis of the orthogonal experimental design, among the three factors that affected the peak value of the contact pressure, the varus angle had the greatest effect, followed by the posterior slope angle, while the external rotation angle had the smallest effect.

**Fig. 6:**
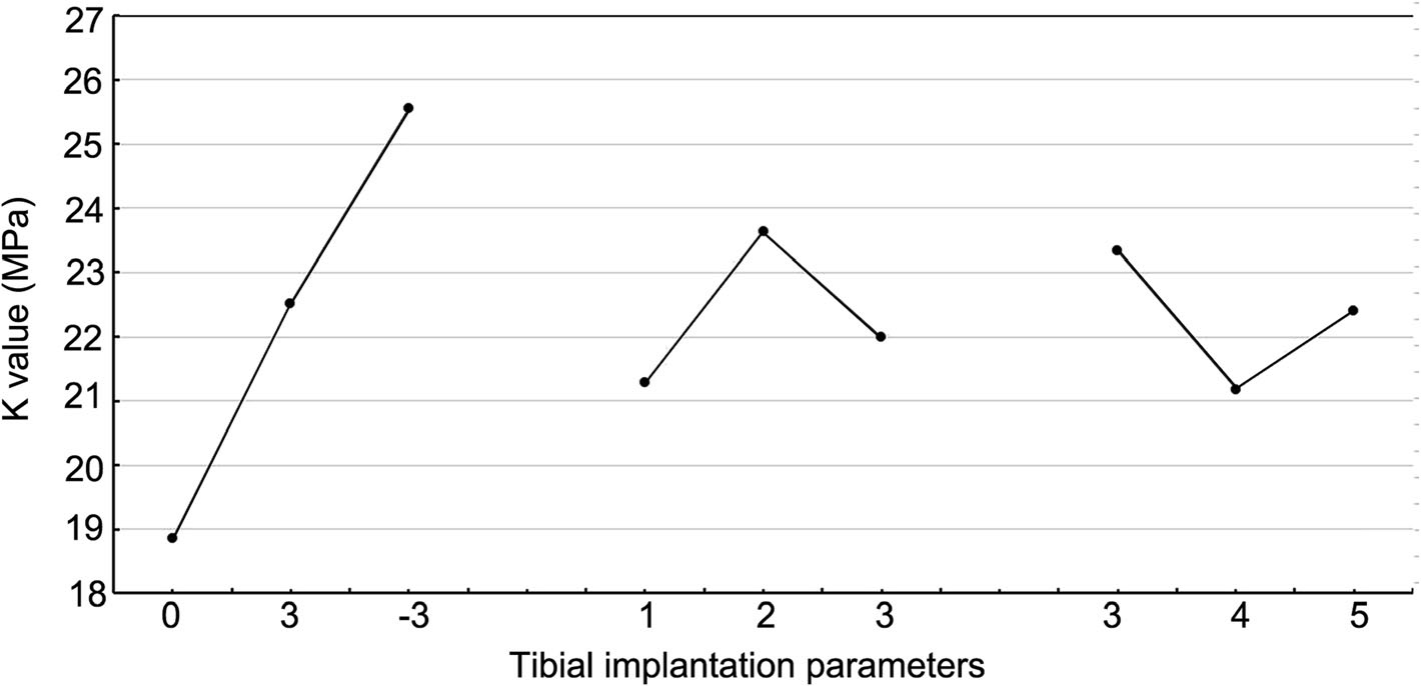
Trend influence of tibial implantation parameters on the peak value of the contact pressure on the polyethylene liner

Through the range analysis, the trend chart on the influence of different tibial implantation parameters on the peak value of the contact pressure was obtained (Fig. 6), and the optimal implantation parameters for the tibial prosthesis were obtained as A_1_B_1_C_2_, which were 0° varus, 1° posterior slope, and 4° external rotation.

### Verification of the optimized implantation parameters derived from the orthogonal experimental design

To verify the optimization results of the orthogonal experimental design based on the optimal tibial prosthesis implantation parameters, the TKA knee finite element model was re-adjusted to carry out finite element analysis. The results showed that the peak value of the contact pressure on the polyethylene liner was 16.37 MPa (group A_1_B_1_C_2_, Fig. 5j), which was located in the medial compartment of the TKA knee. The peak value of the contact pressure was lower than that of the other models and was reduced by 13.36 MPa from the highest value of 29.73 MPa (45.4%). The difference of the peak value of contact pressure between the medial and the lateral compartments was only 0.62 MPa, and the load was more uniformly distributed.

## Discussion

Through optimizing individualized TKA prosthesis implantation parameters, neutralized mechanical alignment of the lower extremity can be restored, promoting uniform load distribution of the medial and lateral compartments of the knee joint, reducing wear, and extending the survival of the prosthesis [16]. Based on the finite element analysis and orthogonal experimental design, the optimal implantation parameters of individualized TKA tibial prosthesis for the knee joint were investigated in this study and showed that 0° varus, 1° posterior slope, and 4° external rotation were the optimal values. The load distribution was uniform in both the medial and lateral compartments, the peak value of the contact pressure on the polyethylene liner remained the lowest, and the wear of the polyethylene liner was reduced. At the same time, the results of this study showed that among the three factors that affect the peak value of the contact pressure on the polyethylene liner, the varus angle showed the greatest effect, followed by the posterior slope angle, and the external rotation angle showed the smallest effect. These results showed that the tibial prosthesis should be accurately implanted during TKA to obtain 0° coronal alignment of the lower extremity, and the appropriate posterior slope angle of tibial prosthesis should also be required.

Although TKA achieves great success, the one-year satisfaction rate after TKA is only about 80% [2]. It has been reported that the surgical error is the most common cause of postoperative failure of TKA [17]. It is a common technical problem to lose the neutral alignment of the lower extremity due to improper implantation of the TKA prosthesis. How to accurately implant the prosthesis, reduce surgical errors, and improve the therapeutic efficacy of TKA are serious problems faced by orthopedic surgeons. Detailed preoperative planning assists in optimizing the implantation accuracy of the prosthesis, improving the therapeutic efficacy of TKA. The most commonly used preoperative planning method in clinical practice is by determining the TKA implantation parameters through lower limb imaging data measurement [6]. In this study, individualized TKA implantation parameters were determined through finite element analysis combined with orthogonal experimental design, which in turn effectively improves the implantation accuracy of the prosthesis, reduces surgical errors, restores neutral alignment of the lower extremity, visually observes the load distribution in the knee joint, and effectively reduces the peak value of the contact pressure on the polyethylene liner. The individualized optimization method of the tibial implant that was identified in this study is different from that of the conventional preoperative measurements. To our knowledge, there are no similar published studies yet. Nevertheless, despite different research purposes, the TKA model in this study was consistent with that of the contact pressure distribution of TKA models described in the previously published literature. The peak value of the contact pressure of the medial compartment of the varus knee model (including 0° coronal knee model) was higher than that of the lateral compartment, while the peak value of lateral compartment of the valgus knee model was higher than that of the medial compartment [4, 9, 18, 19].

The restored neutral mechanical alignment of the lower extremity after TKA can maintain the uniform distribution of the load in the knee joint, reduce wear and looseness of the prosthesis, and increase the survival of the prosthesis [16, 20–22]. Previous studies showed that the coronal deviation of the mechanical axis of the lower extremity > 3° (varus or valgus > 3°) would reduce the clinical efficacy after TKA [23–25]. To a clinically acceptable range, we set the three levels of varus angle of the tibial prosthesis as − 3°, 0°, + 3° in the orthogonal experimental design in this study. Some studies showed that the maintenance of 0–7° posterior tibial slope in the sagittal plane was beneficial for the long-term stability of the prosthesis [26, 27]. The tibial posterior slope angle of our volunteer was 4°, and the tibial prosthesis selected in our study had its own inherent 3° posterior slope angle, so we set the three levels of the posterior slope angle of the tibial prosthesis in our orthogonal experimental design as 1°, 2°, and 3°. Previous study reported that tibial prosthesis with 2–5° external rotation demonstrated better clinical efficacy and less wear of the polyethylene liner with respect to the posterior margin line of the proximal tibial cut [28–30]. The external rotation angle of the tibial prosthesis must match that of the femoral prosthesis during TKA. The transepicondylar axis was rotated 3° externally with respect to the posterior condylar line in the axial plane on our volunteer, and this external rotation angle of Asian races is often greater than 3 ° [26, 27]. Therefore, we set the three levels of the external rotation angle of the tibial prosthesis at 3°,4°, and 5° in our orthogonal experimental design. In fact, the accuracy of the tibial prosthesis implantation angle is important during TKA, which is supported by the results of our study. The results revealed that although the clinically acceptable tibial prosthesis implantation angle was within the range, different combinations of tibial prosthesis implantation angles lead to great changes in the peak values of the contact pressure on the polyethylene liner. The maximal value of the contact pressure was 29.73 MPa, and the minimum value was 16.37 MPa, with the biggest difference being 13.36 MPa (81.6%). The maximal difference in the peak value of the contact pressure between the medial and lateral compartments was 6.52 MPa. Stan et al. reported that changing the implantation angle of the tibial prosthesis by 6°, i.e., from 3° valgus to 3° varus, in the coronal plane led to 46.3% change in the peak value of the contact pressure on the polyethylene liner [9]. In this study, the same 6° change in the tibial prosthesis implantation angle in the coronal plane could cause an 81.6% alteration in the peak value of the contact pressure, which might be considered as additional alterations in both sagittal and axial planes, amplifying this effect synergistically. Thus, if a significant alteration of the tibial prosthesis implantation angle due to surgical technique error was beyond the clinically acceptable range, the distal articular surface of the femoral component would significantly mismatch with the upper surface of the polyethylene liner, causing a great change in the peak value of the contact pressure, and accelerating polyethylene liner wear.

Most of the previous finite element analyses were used to clarify the effects of different tibial prosthesis implantation parameters on the knee contact mechanics after TKA, which suggested the surgeons be cautious during TKA. Through finite element analysis, the peak values of the contact pressure on the polyethylene liner were 11, 16.1, and 32.31 MPa when the tibial prosthesis was 0°, 3°, and 8° varus, respectively. The higher the varus angle was, the greater the change in the peak value of the contact pressure was, more likely causing polyethylene wear. This suggested that during TKA, orthopedic surgeons should minimize varus implantation of the tibial prosthesis as much as possible [9]. Shen et al. evaluated the effect of four different posterior tibial slopes (0°, 3°, 6°, and 9°) of the tibial prosthesis on the contact pressure of the polyethylene liner, and found that the contact stress at 3° and 6° posterior slopes was almost uniformly distributed in the medial and lateral compartments, and the wear of the polyethylene was the lowest, indicating the importance of proper posterior tibial slope [11]. Osano et al. analyzed the changes in the peak values of the mixed stress on the polyethylene liner when the tibial prosthesis was in neutral position (0°), 15° internal rotation, and 15° external rotation. The results revealed that the peak stress on the polythene liner was increased by 160% when the tibial prosthesis was at 15° internal rotation compared with the neutral position, which significantly increased the wear of the polythene liner. This suggested that the tibial prosthesis should be implanted in the neutral position during TKA through accurate proximal tibial resection [10]. Different from the above studies, the present study analyzed the effect of three different implantation parameters, including the varus angle (− 3°, 0°, and 3°), the posterior slope angle (1°, 2°, and 3°), and the external rotation angle (3°, 4°, and 5°), on the contact behavior of TKA knee joint to determine the optimal implantation parameters of tibial prosthesis by an orthogonal experimental design for preoperative planning, so the tibial prosthesis could be more accurately implanted during TKA. The optimal implantation parameters of the tibial prosthesis found in this study included 0° varus, 1° posterior slope, and 4°external rotation. As the tibial prosthesis used in this study was designed with an inherent posterior slope of 3°, the optimal implantation parameters of tibial prosthesis actually included 0° varus, 4° posterior slope, and 4°external rotation. At this time, the peak value of the contact pressure on the polyethylene liner remained the lowest, with 16.37 MPa. It also revealed that, within the clinically acceptable value range, the varus angle has the greatest effect on the peak value of the polyethylene liner, followed by the posterior slope angle and the external rotation angle. It is likely that the varus and the posterior slope have a great effect on the matching degree between the upper surface of the polyethylene liner and the distal articular surface of the femoral prosthesis when compared with the external rotation angle. In addition, the peak value of the contact pressure on the polyethylene liner was larger, and the wear effect was more significant than that of the external rotation angle. Therefore, the optimal varus angle and posterior slope of tibial prosthesis should be preferentially restored during TKA; otherwise, non-uniform load distribution and wear of the polyethylene liner would be caused.

In this study, an orthogonal experimental design was selected to reduce the number of TKA knee models, improving their efficiency. A total of 27 TKA knee joint finite element models were needed to include the three implantation parameters under three different levels in this analysis. The orthogonal experimental design significantly improved the efficiency of analysis, and only 10 models assisted us in predicting the distribution characteristics of the contact pressure on all polyethylene liners, saving time as well as in implementing effective TKA preoperative plan. The influence of different implantation parameters on the peak value of the contact pressure on the polyethylene liner could also be determined by the orthogonal experimental design for improving the efficiency of finite element analysis to guide in the accurate implantation of the tibial prosthesis, which has not been reported yet.

This study also has some limitations. First, this study simulated the change of the contact pressure on the polyethylene liner under a static condition of 0° flexion position. The contact behavior of the TKA knee joint under different knee flexion angles or under dynamic conditions should be further investigated to obtain more reliable results in the future. Second, in this study, the previously verified normal knee joint model was used to implant the TKA prosthesis for calculation and analysis. If the knee model of end-stage osteoarthritis was selected for finite element analysis of TKA prosthesis implantation, the analysis results might be closer to that of the actual situation. Third, in this study, the varus angle changed 3° for each level, but the other factors (posterior slope and external rotation) changed by only 1°, which leads to an increase in the mismatch between the distal articular surface of the femoral prosthesis and the upper surface of the polyethylene liner in the coronal plane, which may increase the effect of the tibial varus angle on the peak value of the contact pressure on the polyethylene liner. In future studies, we will set varus angle changes of 1° for each level, similar to the posterior slope angle and external rotation angle, to observe the effect of the three tibial prosthesis implantation parameters on the peak value of the contact pressure. Finally, this study focused on the implantation of TKA prosthesis in the knee joint, without considering the release of medial and lateral collateral ligament and soft-tissue balance. Future studies should further explore the influence of soft tissue balance on the contact behavior in the TKA knee joint, as well as the impact on the polyethylene wear.

Above all, the individualized accurate implantation of TKA tibial prosthesis was optimized based on the finite element analysis combined with an orthogonal experimental design, minimizing the peak value of the contact pressure on the polyethylene liner and reducing the wear. This method has clearly confirmed the effect of different implantation parameters on the peak value of the contact pressure on the polyethylene liner, cautiously guiding the tibial prosthesis implantation during TKA. Even minor changes in tibial implantation parameters can lead to significant alterations in the peak value of the contact pressure on the polyethylene liner, affecting the long-term life of the prosthesis. The tibial prosthesis should be accurately implanted during TKA, and appropriate tibial prosthesis varus and posterior slope angles should be selected.

## Conclusions

In this study, we used a new method of finite element analysis combined with an orthogonal experimental design to optimize the implantation parameters of the tibial prosthesis and minimize the peak value of the contact pressure on the polyethylene liner in order to reduce the wear and prolong the service life of the prosthesis. This method could be adapted for all patients receiving primary TKA, and it could be a new exploration for the preoperative planning of TKA and provide a basis for the biomechanical decision of accurate implantation of TKA prosthesis.

## Data Availability

The datasets used and/or analyzed during the current study are available from the corresponding author on reasonable request.

## Abbreviations

TKA: Total knee arthroplasty
3D: Three-dimensional
